# Should I pursue a PhD in radiology? Opportunities, pitfalls, and practical strategies

**DOI:** 10.1186/s13244-026-02457-4

**Published:** 2026-11-12

**Authors:** Stijntje Willemijn Dijk, Marion Smits, Savvas Andronikou

**Affiliations:** 1 Department of Radiology and Nuclear Medicine, Erasmus MC University Medical Center, Rotterdam, The Netherlands; 2 Department of Epidemiology, Erasmus MC University Medical Center, Rotterdam, The Netherlands; 3 Department of Radiology, Elisabeth-Tweesteden Ziekenhuis, Tilburg, The Netherlands; 4 Brain Tumor Centre, Erasmus MC Cancer Institute, Rotterdam, The Netherlands; 5 Medical Delta, Delft, The Netherlands; 6 Department of Pediatric Radiology, Children’s Hospital of Philadelphia, Philadelphia, PA USA; 7 Department of Radiology, Perelman School of Medicine, University of Pennsylvania, Philadelphia, PA USA

**Keywords:** Research, Continuing medical education, Postdoctoral training, Radiology, Radiography

## Abstract

**Abstract:**

Undertaking a PhD in radiology represents a pivotal career choice: one that can open pathways to international mobility, interdisciplinary collaboration, and scientific discovery. This opinion piece examines the opportunities and challenges of pursuing doctoral training in radiology and provides guidance for medical students, radiographers, residents, and practicing radiologists considering this academic path. Yet the process also entails risks: unrealistic goals, inadequate mentorship or institutional support (such as protected time for research, administrative support, and financial resources), and limited genuine motivation can hinder progress and satisfaction. In this expert opinion article, we highlight the natural synergy between clinical radiology and research skills. Beyond advancing academic credentials, a PhD cultivates competencies in statistics, leadership, and teamwork: skills that support careers across academia, healthcare, industry, and policy. We discuss different PhD formats, from monograph-based to publication-based structures, and offer practical advice on project selection, funding strategies, and supervisory relationships. We conclude with an overarching checklist with a compilation of questions to answer the question “Should I pursue a PhD in radiology?.” By addressing both pragmatic and personal aspects, including mental well-being, this paper provides a balanced roadmap for (future) imaging professionals aiming to design meaningful and sustainable clinical research careers.

**Key Points:**

**Question** Deciding whether to pursue a doctoral degree in radiology requires navigating complex trade-offs between clinical workloads, personal motivations and diverse program formats.

**Findings** Successful doctoral training relies on aligning personal curiosity with realistic project scoping, accessible methodological support and careful supervisor selection. On the other hand, valuable alternative career paths and potential obstacles should also be considered before taking up the PhD challenge.

**Critical relevance statement** Aligning personal goals, skills, and career planning with the institution's expectations can help (future) imaging professionals transition into sustainable research careers.

**Graphical Abstract:**

**Figure:**
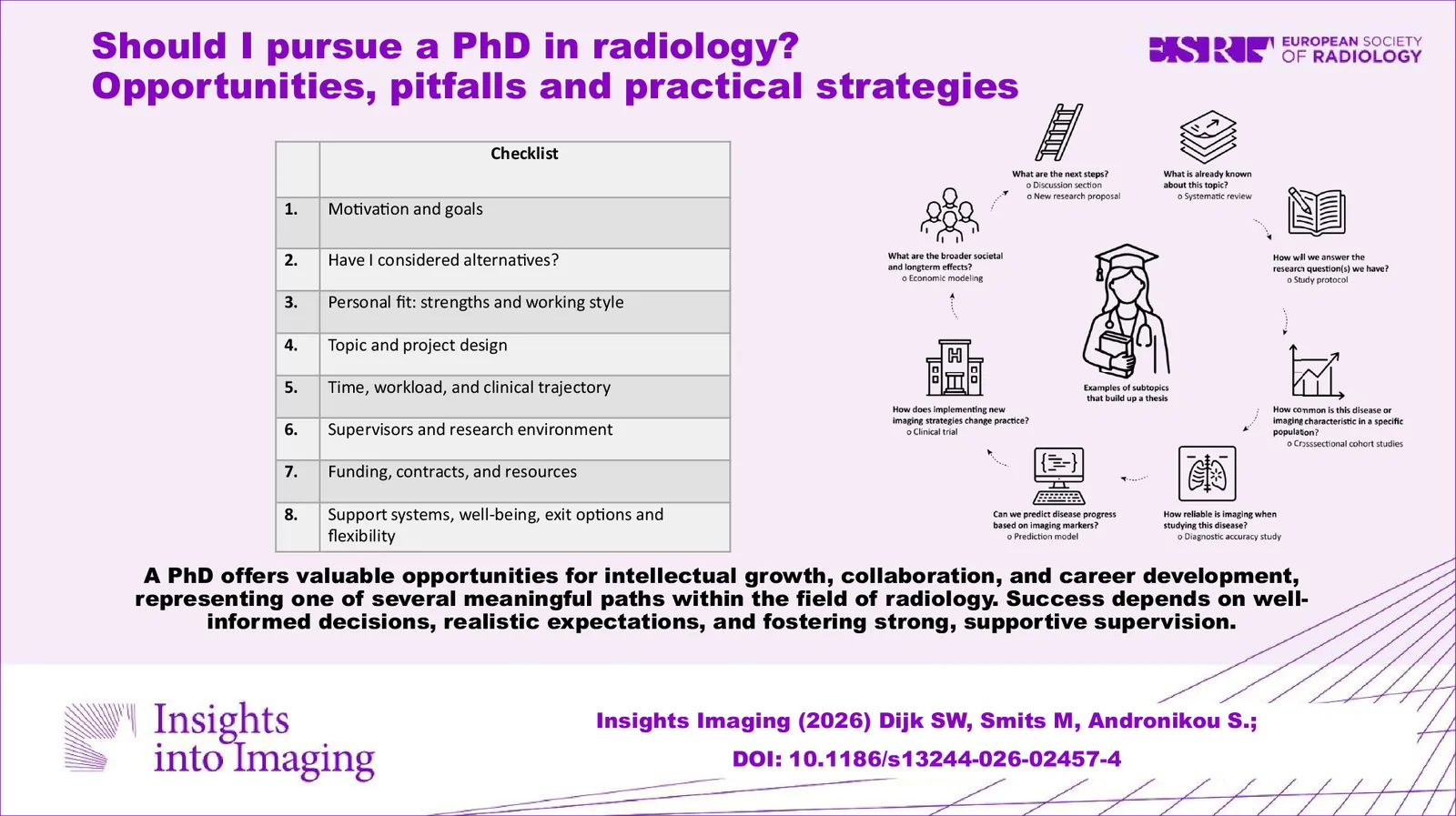

## Introduction—Why talk about a PhD in radiology now?

The pursuit of a PhD in radiology, whether as a (future) radiologist or radiographer, is an increasingly relevant consideration in a landscape where academic output serves as both a professional currency for promotion and an international passport for career mobility. This consideration becomes especially relevant when a specific career seems unattainable without one [1–3]. The value of this degree extends beyond the individual, creating a bridge between scientific inquiry and clinical reality, and integrating evidence-based medicine with clinical decision-making, ultimately enhancing patient care [4, 5]. By integrating routine clinical radiology habits such as pattern recognition and hypothesis generation into formal study design, the radiology researcher is uniquely positioned to ensure that academic work remains grounded in clinical and patient-centered needs.

A diverse group of imaging professionals now explores the MD-PhD path, ranging from medical students and residents to (experienced) radiographers and radiologists seeking a leadership position or academic recognition. This interest is further accelerated by the rapid evolution of artificial intelligence, quantitative imaging, and evidence-based policy, all of which require the methodological grounding and mentorship structure that doctoral training can provide. While the proportion of (future) imaging professionals holding a PhD varies by country and institution, the degree is increasingly viewed as a foundational driver’s license that marks the starting point of an independent research line. Ultimately, the PhD experience is highly heterogeneous, with formats ranging from traditional monographs to contemporary publication-based tracks [6] that may better suit a clinician’s lifestyle. Success requires commitment and careful planning to mitigate the challenges of research.

The aim of this paper is to provide a balanced overview of the opportunities and challenges of doctoral training in radiology. As an expert opinion article, it combines the available literature with practical experience to address issues such as mentorship, funding, and project selection, with the goal of helping imaging professionals decide whether, when, and how a PhD can support a meaningful career. We conclude with an overarching checklist in Table 1 with a compilation of questions to answer the question “Should I pursue a PhD in radiology?”.

**Table 1:** Checklist: Should I pursue a PhD?

			Yes	No	Not sure (yet)	Not applicable
1.	**Motivation and goals**					
		I am primarily motivated by curiosity and improving patient care, not just the PhD title, or because “everyone is doing one".	☐	☐	☐	☐
		I enjoy thinking about research questions, methods, and the interpretation of data.	☐	☐	☐	☐
		I like writing, and I am willing to learn and invest in becoming better at scientific writing.	☐	☐	☐	☐
		I can imagine still being interested in my topic or method area several years from now.	☐	☐	☐	☐
		I have at least a rough idea of how a PhD could support my long-term career vision (academic, clinical leadership, industry, policy, etc.).	☐	☐	☐	☐
		I understand that a PhD is training to become an independent researcher, and that aligns with my goals	☐	☐	☐	☐
		I am prepared for delayed rewards (papers, thesis, degree) and not just short-term gratification.	☐	☐	☐	☐
		I know that a PhD does not guarantee an academic career nor is it mandatory for being a good clinician, and I am okay with that.	☐	☐	☐	☐
2.	**Have I considered alternatives?**					
		I have seriously considered, or previously tried out doing smaller research projects (e.g., single papers, QI projects, audits) without committing to a full PhD and considered the pros and cons of both.	☐	☐	☐	☐
		I have explored whether a master’s degree, courses, or shorter research tracks could meet my goals.	☐	☐	☐	☐
		I have considered the opportunity cost of committing to the PhD, and the other ways of “going beyond clinical work” (teaching, guideline work, advocacy, curriculum development, etc.)	☐	☐	☐	☐
		After thinking about these alternatives, a PhD still feels like the most fitting route for my goals and preferences.	☐	☐	☐	☐
3.	**Personal fit: (aspired) strengths and working style**					
		I am comfortable with long-term projects that may take several years to complete.	☐	☐	☐	☐
		I can break big goals into smaller tasks and keep going despite slow progress.	☐	☐	☐	☐
		I am willing to tolerate uncertainty (e.g., data delays, revisions, rejections).	☐	☐	☐	☐
		I can handle constructive criticism of my work and use it to improve.	☐	☐	☐	☐
		I recognize tendencies like perfectionism or procrastination in myself and am willing to work on them so they do not paralyze progress	☐	☐	☐	☐
		I am prepared to learn or strengthen skills in statistics, methods, and project management.	☐	☐	☐	☐
		I am open to collaboration and teamwork, as well as working alone for longer periods of time.	☐	☐	☐	☐
		I can ask for help when I am stuck (statistics, writing, methods, mental health).	☐	☐	☐	☐
4.	**Topic and project design**					
		I have or can identify a topic area that connects to real bottlenecks or unmet needs in clinical practice, guidelines, or patient priorities.	☐	☐	☐	☐
		I understand that a PhD does not have to be built on a single “genius idea” but can be a coherent set of feasible studies.	☐	☐	☐	☐
		I can see a plausible path to several publishable subprojects (e.g., retrospective PACS study, outcomes research, protocol optimization, health economics, etc.) based on available or realistically obtainable data	☐	☐	☐	☐
		I am willing and able to balance ambition with feasibility rather than chasing an overly grand or unfocused project.	☐	☐	☐	☐
5.	**Time, workload, and clinical trajectory**					
		I have a realistic estimate of how many hours per week I can devote to PhD work over several years.	☐	☐	☐	☐
		I know and have discussed whether this PhD will be: full-time, part-time alongside clinical work, or “in my free time” on top of existing duties.	☐	☐	☐	☐
		If part-time or alongside residency/clinical work: I have a clear plan for when and where I will work on my PhD (not just “whenever I find time”).	☐	☐	☐	☐
		If part-time or alongside residency/clinical work: I have discussed with relevant leaders (program director, head of department) how a PhD will interact with my clinical training and performance expectations.	☐	☐	☐	☐
		I have considered the different timepoints in my career, and I could pursue a PhD (e.g., during/after medical school, before/during/after residency, and I have considered the pros and cons of each of these and believe this current point in time is a good fit for me.	☐	☐	☐	☐
6.	**Supervisors and research environment**					
		I have identified at least one potential supervisor with relevant content or methodological expertise.	☐	☐	☐	☐
		I have checked that this person and/or team have a track record of successful PhD completions and satisfied graduates, where possible.	☐	☐	☐	☐
		I have talked to current or former PhD candidates in this group (if possible), and their experiences do not raise major red flags.	☐	☐	☐	☐
		I have some evidence that my potential supervisor(s) are accessible (e.g., respond to emails, schedule meetings, provide feedback in a reasonable time). For example, by working with them previously on smaller projects, by hearing from others in the department, or other forms of evidence that I deem representative.	☐	☐	☐	☐
		I have openly discussed expectations about topics including but not limited to: meeting frequency, turnaround time for feedback, authorship and contributions, teaching/clinical duties, level of supervision versus autonomy, departmental duties outside of the scope of my thesis, conference attendance and coursework, expected output including numbers of papers, research time arrangements, presence at the office/lab/department versus working from home, working hours	☐	☐	☐	☐
		I feel I can communicate honestly with my potential supervisor(s), including if I am struggling.	☐	☐	☐	☐
7.	**Funding, contracts, and resources**					
		I understand the main funding models and options in my context, such as funded project-based positions, clinical-academic combined contracts with protected time, personal fellowships, unfunded/partially funded trajectories alongside a clinical salary. I know which model my potential PhD falls under.	☐	☐	☐	☐
		I have a realistic plan for my own income during the PhD (salary, stipend, maintaining clinical work, income generated by teaching, etc.).	☐	☐	☐	☐
		I know who pays for key project-related costs (imaging, data management, software, statistics, etc.), or I know how these will be covered.	☐	☐	☐	☐
		I have checked the local requirements (entry requirements, number of papers, journal quality, coursework, defense rules, deadlines, whether the thesis is monograph-based or publication-based).	☐	☐	☐	☐
		I have or will have written agreements (or at least clear emails) documenting: supervision expectations, access to data and facilities, research time arrangements, expected outputs, coverage of coursework, conferences or other expenses, start and end dates of the PhD, options of extending the PhD trajectory when necessary, and the conditions under which I will be able to defend my thesis. I am comfortable starting this PhD under these funding and contract conditions, or I will not start until conditions are acceptable.	☐	☐	☐	☐
8.	**Support systems and well-being, exit options and flexibility**					
		I have, or can build, a peer network (other PhD students, local research groups, online communities) to normalize challenges and share experiences.	☐	☐	☐	☐
		I have or know how to find an independent mentor who can guide me through academic setbacks, advocate for my professional growth, and provide an objective perspective.	☐	☐	☐	☐
		I am aware that setbacks (rejections, delays, changing analyses) are normal and not a sign of personal failure.	☐	☐	☐	☐
		I have thought about how I will maintain my mental health (sleep, exercise, hobbies, family/social time) alongside PhD work.	☐	☐	☐	☐
		I know where to seek additional support if needed (occupational health, counseling, resilience resources, institutional support).	☐	☐	☐	☐
		I have discussed my plans with key people in my personal life (partner, family, close friends) and considered their reactions and needs as well as my own.	☐	☐	☐	☐
		I could accept, in principle, that if key conditions change dramatically (supervision breaks down, life crisis, major misfit), it would be legitimate to reconsider continuing. I accept that, in rare cases, changing topic, supervisor, or even stopping a PhD can be the healthiest option. I accept that protecting my health is more important than stubbornly finishing at any cost.	☐	☐	☐	☐
		I understand that even an unfinished PhD can still yield transferable skills (data handling, writing, collaboration, critical thinking). I have thought through what “success” would look like for me, in terms of skills gained, networks built and impact on patient care.	☐	☐	☐	☐

Read each item and answer “yes”, “no”, “not sure (yet),” or “not applicable” honestly

**How to use this checklist**

After completing the checklist, look at your pattern of answers and weigh both the changeable conditions and the realistic alternatives: if most items are “yes”, a PhD is likely a good option; if many are “no”, first ask whether those “no’s” could be changed by modifying the environment (for example, finding a different supervisor or group, negotiating protected time, or choosing a more feasible topic) before concluding that a PhD is not for you. When many answers are “not sure yet”, identify what concrete information is missing and seek it out deliberately, for instance, by speaking with people in your department, radiologists who have completed a PhD, or contacts you meet at courses and conferences. If, after this exploration, important barriers remain (e.g., persistent lack of supervision, no viable time or funding arrangements, or a mismatch with your preferred working style), consider postponing your decision, starting with smaller research projects, or investing in alternative forms of scholarly work until the foundations for a sustainable PhD trajectory are stronger

## The synergy between clinical radiology and research

For those wondering whether a career in imaging translates well to their daily practice, we believe there are many similarities between the activities and skills involved in clinical radiology and research. Students who have completed an MD-PhD program are more likely to choose a career in radiology than, for example, psychology, anesthesiology, surgery and rehabilitation, obstetrics, pediatrics, emergency or family medicine [7]. Far from being an isolated world of theory, research is, in many ways, a natural extension of the cognitive toolkit a radiologist uses in everyday practice.

Every case requires pattern recognition, problem diagnosis, and the ability to draw inferences from incomplete information. Radiologists are trained to connect the dots across clinical data points, a skill set that also forms the backbone of scientific inquiry. While clinical work focuses on the individual patient, research extends these traits into formal study design, statistics, and the construction of coherent arguments that can influence thousands.

Radiology possesses a uniquely interdisciplinary and translational character at the intersection of clinical medicine, physics, engineering, computer science, and data science. This allows the radiologist-researcher to act as a bridge, translating research questions into clinical and real-world patient benefits. By leveraging an aptitude for analytical thinking and a healthy dose of curiosity, those in radiology are uniquely equipped to lead research that transforms bottlenecks in daily practice into new and relevant solutions. A PhD offers a structured framework, pathway and guidance to transforming these daily observations into a proper and valid study.

## Why pursue a PhD, and what are the potential benefits?

A PhD can contribute to radiology’s long-term development by generating knowledge that helps shape future practice and patient care. For many candidates, the appeal of a PhD lies not only in its scientific output but also in its personal and professional rewards [8]. It stimulates ideas, fosters collaboration, and builds networks that can enrich a radiologist’s career for years to come. Working with multidisciplinary teams and across institutions or countries often leads to lasting professional relationships. It also strengthens transferable skills such as critical appraisal, statistics, scientific writing, presenting, project management, leadership, and collaboration, all of which are valuable in academia, clinical leadership, industry, policy, consulting, and other non-clinical careers [3]. In some health systems, a PhD is expected or even required for academic, consultant, or leadership roles. It can also improve mobility across countries and sectors, including industry, regulatory agencies, and health technology assessment or health economics roles. In addition, it provides formal recognition of research expertise. The process often includes publishing several papers and contributing to professional societies or guideline groups, which strengthens a CV and helps establish national and international academic, leadership and/or subspeciality visibility.

A PhD differs from other forms of research involvement in that it is designed to train an independent researcher who can define, lead, and sustain a coherent line of inquiry rather than contribute mainly in a supporting role. It can also open the door to sub-specialization, mentorship and leadership, allowing the holder to help shape the next generation of researchers and, in time, influence the direction of the field itself [8].

## Reasons *not* to pursue a PhD (or not now)

Before starting a PhD, candidates should consider whether they are drawn to the idea of doing research itself or primarily to the degree. There is an important distinction between enjoying research and (feeling) a need to obtain the formal PhD title. Many valuable forms of academic engagement, including research participation, quality improvement, and scientific writing, can be pursued without enrolling in a doctoral program. For some individuals, this may be a better first step to explore whether the type of work suits them. A PhD is typically expected to prepare someone to design, lead, and complete an independent research line, whereas clinicians primarily interested in identifying relevant questions, prioritizing problems, or contributing clinical insight may find a consultative role more fitting. In practice, these roles are often complementary: researchers may primarily provide methodological expertise, while clinicians contribute the clinical perspective and help define the questions that matter most, a role that may also be more easily combined with clinical responsibilities. Additionally, a PhD is not necessarily a guarantee for a future research career [9]. For example, in the Netherlands, the percentage of PhD graduates without research output was 41.9% (78/186) at 5 years, 28.6% (24/84) at 10 years, and 16.1% (5/31) at 15 years in the post-PhD period [10].

Starting with a smaller research project can also be a useful way to gain experience in scientific thinking, analysis, and collaboration before committing to a multi-year PhD. This may help determine whether the topic, the working style, and the supervisory team are a good fit. It is also worth remembering that a PhD (or research in general) is not the only way to contribute meaningfully beyond routine clinical work. Other valuable academic and professional activities include clinical audits, collaborative departmental or societal projects, supervision of master’s theses, teaching, communication, patient information development, advocacy, and work on guidelines or policy. For individuals who prefer practical, problem-solving work, these alternatives may offer more gratifying immediate and concrete impact than a PhD.

A PhD can be a rewarding path, but it is not the right choice for everyone at every stage of their career. When the fit is poor, it may lead to frustration, anxiety, or unfinished work, resulting in a loss of time and resources for both the PhD candidate and those around them. Common reasons for dissatisfaction include a mismatch between project demands and personal preferences, such as difficulty with long-term single-goal work or underestimating the time required, as well as poor supervision, unclear expectations, and a lack of support. Pursuing a PhD mainly for status is a weak foundation and may result in limited scientific value of the work being done, and personal dissatisfaction. Importantly, a PhD alone does not guarantee clinical excellence or suitability for a residency, subspecialty or consultant role, even though, in some places, it used to be considered the only way to obtain such a position.

Helpful motivations in contrast include curiosity, a genuine wish to improve patient care, enjoyment of analysis and writing, and interest in academic or leadership roles. High-risk motivations, on the other hand, include external pressure, prestige, or the belief that “everyone” needs a PhD. In that sense, the decision is less about whether a PhD is valuable in general and more about whether it is the right step for the individual, at the right time.

## Is the PhD experience for me?

There is no singular PhD experience. Expectations, structure, and requirements vary widely between institutions and countries, so a program that suits one candidate may be unattractive to another. In some settings, such as the U.S. or the Netherlands [11], a PhD follows a traditional model with coursework, regular on-campus attendance, progress meetings, and a final thesis submission and defense. This format often culminates in a monograph-style dissertation with integrated chapters covering the literature review, methods, results, and discussion. A PhD may also be integrated into a combined medical and PhD program, such as the MBBS/PhD program in Singapore, the MBChB/PhD program in South Africa [12].

Other programs are more flexible. For example, some countries may not require additional coursework, such as Japan or Germany [11]. They may offer research support without requiring formal classes or frequent campus visits, and supervision may occur largely online or be more sporadic. In some places, such as Australia, an oral defense may not be required to obtain a PhD [11]. In such systems, the degree may be obtained through a publication-based route, in which the thesis consists of a series of peer-reviewed papers accompanied by an overview and integrative discussion. Requirements such as the minimum number of papers, journal reputation, and timing of the defense differ by institution and country. This latter format may be chosen more frequently by imaging professionals intending to combine their PhD with clinical work, whereas the former may be more common among those pursuing a PhD full-time.

## What kind of radiology research project can I do, and what about subject matter?

Common examples of PhD projects include clinical imaging, interventional radiology, workflow research, epidemiology, outcomes research, health services and health economics/technology research, imaging markers, radiology-pathology correlation and technical development or protocol optimization. A practical way to choose a topic is to focus on an area one already knows well, or one in which one wants to develop deep(er) expertise.

Not all PhD research involves a new invention, and not all projects require an experiment. In some cases, a PhD may add new evidence without being entirely novel in the strictest sense, for example, when replicating earlier studies.

Research questions often arise from clinical bottlenecks and unmet needs observed in daily practice. Other sources include priorities identified by patient organizations or professional societies, knowledge gaps highlighted in guidelines or systematic reviews, and health economic or value-of-information analyses showing where additional evidence could change decisions [13]. Existing projects can also be extended into a PhD by building on earlier work and developing it into a coherent set of studies.

In some settings, particularly funded PhD tracks or standard project-based positions, the candidate has limited choice in topic because the PhD forms part of a larger research program. This can be an advantage for those who want to learn a specific method or enter a new field, but it may feel restrictive for candidates who want more freedom to shape their own research direction.

**Fig. 1:**
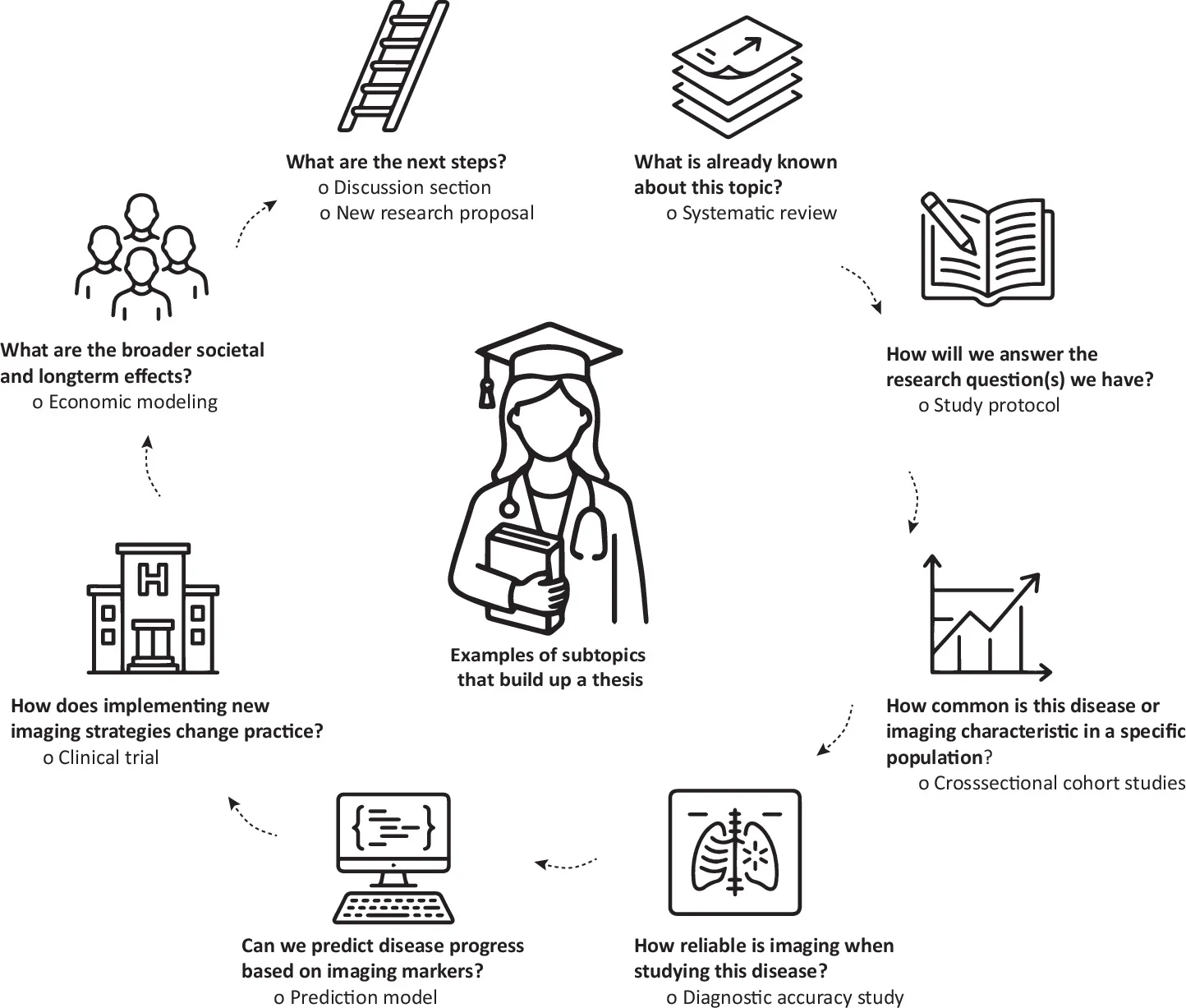
Example subtopics for planning multiple papers on a disease, to form a publication-based PhD

For practicing imaging professionals, many worthwhile research questions emerge directly from clinical practice. These questions can often be developed into a structured series of subprojects, each suitable for publication and together forming a thesis that is well-suited to a PhD via publication. Figure 1 is one example of developing multiple clinical publications from a single clinical disease or dataset around a specific topic. The availability of large retrospective PACS datasets also makes data collection more feasible than in the past, whether the work involves reinterpreting images, reanalyzing existing data or using radiology reports.

Because radiology sits at the intersection of medicine, physics, and computer science, it offers a huge variety of directions for a PhD. Candidates may hesitate to start because they feel they haven’t found that “one topic” or specific disease they are deeply passionate about. While having a lifelong interest in a particular pathology is one way to stay motivated, it certainly isn’t the only path.

Another driver can, for instance, be the methodology, mastering a specific type of decision modeling [13] or a new imaging technique that can be applied to many different clinical problems. Alternatively, the topic might be less important than the working environment. Choosing a project based on a supportive supervisor, a strong team, and reliable access to data can at times be a better move than chasing a specific topic in a vacuum. Ultimately, the PhD can serve as a springboard. The skills one gains from building a project from scratch are transferable to other medical fields or even to other industries. The project should fit the available time, funding, data access, and technical resources, while also balancing innovation with feasibility.

## PhD supervision

The quality of supervision is one of the strongest determinants of PhD satisfaction, productivity, and overall well-being. Selecting an appropriate supervisor is, therefore, among the most important and controllable decisions a candidate will make. Effective supervisors are accessible, provide constructive and timely feedback, set realistic expectations, and show genuine interest in the candidate’s development and career plans.

Working with a single supervisor can sometimes simplify communication and speed up manuscript approvals, but building a supervisory team with complementary roles may be more beneficial. A balanced team might include clinical experts, methodologists or statisticians, and advisors in research ethics or education, all supporting different aspects of the candidate’s training and project execution. For example, in the North American PhD model, candidates usually have a committee comprising a supervisor and an additional number of faculty members, whereas in the common European model, PhD candidates tend to be accepted into a supervisor’s research group, and the supervisors themselves are responsible for the thesis trajectory [9]. In countries such as South Africa where radiology PhD graduates are few, the choices of radiologist PhD supervisors are  limited, and clinicians with PhDs fill the role, bringing research expertise and clinical content expertise, while the addition of non-PhD radiologists with specific content expertise to the support team as co-supervisors provides radiological insight.

To avoid misunderstandings and manage expectations, clear agreements should be made from the start regarding meeting frequency, feedback turnaround time, authorship, and division of responsibilities. Candidates should remain alert to potential pitfalls, such as supervisors who (intentionally or unintentionally) delay submission, insist on excessive control over wording, or have specific (competing) personal interests that limit publication opportunities. Conversely, overly hands-off supervisors may offer too little guidance, risking stagnation and repeated journal rejections.

When seeking a supervisor, candidates can start within their own department or explore opportunities in other hospital units, universities, national societies, or international networks such as the European Society of Radiology  (ESR) (Table 2 below). Conferences and professional courses can provide excellent opportunities to identify potential supervisors. Initial communication should be concise, tailored, and show a clear interest in the supervisor’s work, preferably with a concrete suggestion for collaboration, such as a short student project. Additionally, there may be an explicit vacancy for a junior researcher within an ongoing research project, in which case one should carefully consider what the team is looking for in a PhD candidate and how that fits with one’s own capacities and ideas. The vacancy need not be a perfect fit; one may not yet have certain skills but be willing to learn them, or the topic may not be immediately aligned with original goals, but there may be space to expand the research question into additional sub-questions that provide a better fit. In departments with an established research structure and culture, selecting supervisors with a proven record of successful completions and satisfied PhD graduates is often the safest and most productive choice.

**Table 2:** Tips for finding a research mentor and PhD supervisor

Some tips for finding a supervisor include:
(a) Looking within the home department, other hospital departments, universities, national societies and international networks (like ESR).
(b) Use of courses, conferences and research schools to meet potential supervisors.
(c) Practical tips for first contact: concise emails, reference to specific work (not some generic I like your work, I want to work with you), clear interest, and proposal for a small initial step (e.g., student project).
(d) If there is already a PhD/research culture in a department, with multiple options for supervision, choose supervision that has a track record of successful completion and satisfied candidates.

## Funding, resources, and contracts

Funding can play an important role in many PhD trajectories, supporting salaries, project execution, patient recruitment, imaging examinations, equipment use, and administrative needs. Salary support may come from several sources:

- Funded PhD tracks, in which the candidate holds a project-based position financed by a grant, usually under an established principal investigator; these often are considered *project-grants*
- Clinical-academic combined contracts, offering *protected research time* within a clinical post; and
- Personal fellowships or scholarships, which the candidate obtains independently. These are often considered *personal career grants*.

Especially personal career grants can be highly competitive, as they facilitate a person to do research in a much freer format, and they are usually awarded to people further along the career ladder or with a particular profile (e.g., to support international exchange or an underrepresented demographic of researchers). Project-based funding, on the other hand, is dedicated to a specific project or research question the funder wants answered, often already has a more senior researcher committed to it, and often seeks a more junior researcher to execute the research.

Alternatively, many unfunded or partially funded PhD trajectories offer a viable, more accessible option. Advantages of not seeking funding may include more (academic) freedom (as there is no funder to report to), flexibility in topic selection and the ability to start the work without going through the grant application (and often rejection) process. For clinical radiologists, radiographers and residents who are already employed in a clinical capacity that provides them with a base salary, this PhD trajectory can be a more accessible solution than a full-time researcher who is dependent on research-based income. Some residency programs may even offer dedicated research time, although there are wide variations to such schemes; for example, even within one country like the U.S., the offer of protected research time can range from zero to 61% of NIH programs in years 1–3 and can include even all residency programs in year 4 [14].

Nevertheless, there are downsides to this route: choosing to do research can add onto the workload, or take away time from clinical education or practice, and one is still dependent on supervisory commitment, which may be less clearly defined without a paid contract and may place one inherently lower on the supervisor’s priority list when there is no financial deadline to finishing the degree.

Written agreements on supervision activities, frequency, access to facilities (such as a journal library or the hospital network), access to data, dedicated research education, time commitments and/or time off from clinical duties and expected outputs should be clarified prior to starting any PhD journey, whether funded or unfunded.

## Getting stuck, setbacks, and moving forward

Highlights and challenges are both a normal part of any PhD journey. Common obstacles include external factors, such as delays in data collection or recruitment, technical failures, the loss of key collaborators, and limited supervisor feedback. Personal difficulties, such as a mismatch between the PhD experience and personal expectations, burnout, or family issues, can also slow progress. Individual characteristics such as procrastination, perfectionism, or a lack of confidence often compound these problems and make completion more difficult. Some of these characteristics, such as perfectionism, are common among healthcare professionals, including radiographers and radiologists [15]. While these can be a great strength for meticulous data handling and paper writing, a multi-year PhD trajectory may make it more challenging for people with these traits to eventually accept that a paper or thesis is finally “good enough” for submission and make it through to the defense.

Progress resumes when large, abstract goals are broken down into smaller, manageable tasks with clear timelines. A publication-based PhD format can be helpful, as it allows milestones to be achieved and celebrated through completed papers rather than working towards one single, final product. Regular meetings with supervisors keep the project dynamic; topics, aims, and analyses can be adjusted as needed, since research is rarely fixed from the start. Seeking additional support is another strategy: help with statistics, participation in writing groups, advice from peers or graduate schools, a mentor (who is, crucially, not part of the PhD project), and even short-term counseling can accelerate progress and relieve stress [16].

Sometimes, the most difficult but best choice may be to change direction. Switching topics or supervisors, or, in certain cases, even deciding not to continue the PhD, are valid and possibly healthy choices worth considering. Protecting mental health and maintaining supportive relationships should take priority over stubborn persistence. Even if a PhD remains unfinished, the experience can still provide valuable transferable skills and perspectives that enhance future professional opportunities [17]. Deciding to quit the PhD trajectory may feel as a failure, but one should realize that continuation also comes at a cost: to one’s own mental health, the opportunity cost of the other possibilities that are let go of, the broader research team who continues to support the project throughout, and society when years of effort are placed in a research project that could have been better allocated. It is important to consider the different factors that come into the struggle: Are they surmountable, can they be tackled by changes in the team, the aims, the organization of the work or other factors surrounding the thesis itself, or is there a true mismatch that cannot, or is not worth the cost of solving? Careful preparation before starting, such as revisiting expectations, supervision agreements, and project feasibility, helps prevent many of these difficulties before they arise (although they can never foresee everything that can follow).

## Recognizing progress and redefining success

PhD training is rarely a linear path. There are periods of rapid progress, punctuated by unexpected pauses, revisions, and obstacles that test a PhD candidate’s (and research team’s) endurance as much as their intellect. Recognizing and celebrating both large and small achievements, such as completing a dataset, submitting a manuscript, attending an important research meeting, or presenting at a conference, helps sustain motivation and provides tangible proof of progress. Defining personal success more broadly also helps. Progress should be measured not only by publications or degree attainment, but also by the competencies and insights gained throughout the journey.

Many successful candidates emphasize the value of building a supportive peer network: sharing drafts, attending local research meetings, and maintaining contact with fellow PhD students can normalize challenges that might otherwise feel isolating [18, 19].

## Conclusion

In summary, a PhD offers valuable opportunities for intellectual growth, collaboration, and career development, representing one of several meaningful paths within the field of radiology. Success depends on making well-informed decisions, setting realistic expectations, and fostering strong, supportive supervision relationships. When these elements align, a PhD can become not only a scientific achievement but also a deeply rewarding stage in personal and professional development.

## Data Availability

Not applicable.
